# Spatial variation and factors associated of solid fuel use in Ethiopia a multilevel and spatial analysis based on EDHS 2016

**DOI:** 10.1038/s41598-023-46897-0

**Published:** 2023-11-20

**Authors:** Jember Azanaw, Mequannent Sharew Melaku

**Affiliations:** 1 Department of Environmental and Occupational Health and Safety, Institute of Public Health, College of Medicine and Health Sciences, University of Gondar, Gondar, Ethiopia; 2 Department of Health Informatics, Institute of Public Health, College of Medicine & Health Sciences, University of Gondar, Gondar, Ethiopia

**Keywords:** Environmental sciences, Environmental social sciences, Medical research, Risk factors

## Abstract

Cooking and heating using solid fuels, such as dung, wood, agricultural residues, grass, straw, charcoal, and coal, is a main source of household air pollution. This indoor combustion encompasses a diversity of health detrimental pollutants, especially for people from low-income countries like Ethiopia since solid fuels are accessible easily at a lesser cost. Limited studies done showing factors affecting in choosing fuel type and no study, which revealed spatial heterogeneity of solid fuel used based on such nationally representative data. Therefore, this study, aimed at investigating spatial variation and determinants of solid fuel use in Ethiopia. This study was done using the data from the Ethiopian Demographic and Health Survey 2016, a national representative sample (16,650) households were included. Spatial and Multi-level logistic regression analysis was done by considering the DHS data hierarchal nature. Variables in the final model with a p-value < 0.05 were reported as significant predictors of using solid fuel. All analyses were done using ArcGIS V.10.7.1 and STATA V.14 software. The finding of this study revealed that 90.8% (95% CI (87.9%, 91.2%)) of households depend on solid fuel for cooking. Based on the final model ;Male household head (AOR 1.38, 95% CI (1.12–1.71)), age of household head (AOR 1.61, 95% CI (1.20, 2.17)), and 1.49 (OR 1.49, 95% CI (1.12, 1.99)) respectively for the age classes of < 30, and 30–40, education attainment no education (OR 3.14, 95% CI (1.13, 8.71)) and primary education (AOR 2.16, 95% CI (2.78, 5.96), wealth index Poorest (AOR 11.05, 95% CI (5.68, 15.78)), Poorer (OR 5.19, 95% CI (5.43, 13.19)), Middle (OR 3.08, 95% CI (2.44, 8.73)), and Richer (OR 1.30, 95IC (1.07, 13.49)) compared to richest, and not accessibility of electricity (AOR 31.21, 95% CI (35.41, 42.67)), were individual-level factors significantly associated with using solid fuel. Community-level factors like households found at large city (AOR 2.80, 95CI (1.65, 4.77)), small city (AOR 2.58, 95% CI (1.55, 4.32)) town (AOR 4.02, 95% CI (2.46, 6.55)), and countryside (AOR 14.40, 95% CI (6.23, 21.15)) compared households found in capital city, community level media exposure (AOR 6.00, 95% CI (4.61, 7.82)) were statistically predictors in using solid fuel for cooking. This finding revealed that a large proportion of households in Ethiopia heavily depend on biomass, especially wood, for cooking. There was a greater disparity on solid fuel use for cooking in Ethiopia. Implementing major policy interventions should be introduced to reduce solid fuel use for cooking and inequalities in accessing clean fuel in Ethiopia.

## Introduction

Energy provision is vital for human survival and an integral aspect of environmental management^1^. The alteration to modern fuels has been slow in most less developing nations, and since high population growth consuming solid fuels for cooking has continued around 2.8 billion since 1990^2^.

Access to clean, affordable, and efficient energy has become a challenge for the majority of households in low to medium-income countries^3^. According to World Health Organization (WHO) estimation, about 3 billion persons use open fire or traditional stoves that are fueled by kerosene and solid fuels^4^. Especially people from low-income countries use solid fuels since these are accessible easily at a lesser price^5,6^. Cooking and heating using fuels such as dung, wood, agricultural residues, grass, straw, charcoal, and coal, is a main source of household air pollution, which is called solid fuel. This indoor combustion encompasses a diversity of health-detrimental pollutants, like particles (complex mixtures of chemicals in solid form and droplets), carbon monoxide, nitrogen oxides, sulfur oxides, formaldehyde, and carcinogens such as benzo[a]pyrene and benzene^7^.

Household air pollution is one of the world’s main environmental and public health problems mainly caused by the use of solid fuels for cooking which includes biomass (e.g., wood, crop residues, animal dung, and charcoal) and coal^8^. Charcoal is also typically used in the household with ineffective stoves and partial burning since there is a poor ventilation system. As a result, carbon monoxide, black carbon, and complex organic carbon compounds produced tremendously high pollutant concentrations in the household^9^. The use of solid fuel remains a continued and greatest commodity for the common inhabitants in Sub-Saharan Africa including Ethiopia^1^.

Household cooking energy can be categorized based on the level of energy development into traditional (firewood, agricultural wastes, etc.), intermediate (charcoal, coal, kerosene, etc.), and modern (solar, liquefied petrol gas (LPG), electricity, etc.). Based on the method of production; they can be classified into primary (from natural resources, e.g., firewood) and secondary (from the transformation of primary energy sources^10^.

The use of such dirty energy sources for cooking and heating presents a thoughtful international health risk due to Indoor air pollution (IAP)^11^. Globally, the most significant direct health risk is the pollution produced by partial burning of solid fuels for cooking, and heating^12^. The health outcome of the comparatively great exposures to particulate matter in households where cooking is take place with less effective and poorly ventilated stoves. The continual use of solid fuels has been related to increased disease and death^13^. According to WHO 2012 report indoor air pollution (IAP) caused 4.3 million premature deaths internationally and outdoor air pollution caused 3.7 million mortality^14^. Exposure to IAP was the second uppermost environmental risk factor in the Global Burden of Disease^15^, with an estimated 1.64 million attributable deaths^16^.

Several studies have shown several socio-economic factors, such as income, education, size and age of the households, ownership, education level, household size, and cooking culture influence household cooking fuel type^17–20^. Understanding the type and the key determinant factors of household cooking energy consumption is important for the design and implementation of effective policies to enhance access to clean cooking fuel types. A study on solid fuel use have been conducted using this data focused at factors associated on it^20^.However, there is little known about the spatial variation of household solid fuel use in Ethiopia with a nationally representative dataset. Hence, the primarily aim of the study was investigating spatial variation of household solid fuel use and predictors which is used for policymakers in reducing problems associated with indoor air pollution.

## Materials and methods

### Data sources and sampling technique

Ethiopia is the second largest population in Africa and the country is under sub-Saharan countries. The country is divided into nine regions and two administrative cities. This research used the secondary data extracted from the Ethiopian Demographic and Health Survey, 2016, a nationally representative cross-sectional study. The data collection period was from January 18, 2016, to June 27, 2016. The sample frame used in the survey (EDHS, 2016) was stratified into urban and rural domains and further into regions and districts to get an adequate representation of each stratum. A total of 16,650 primary sampling units, including 11,418 rural and 5232 urban, were selected to analyze the type of fuel used among the households. Data were collected from January 18, 2016, to June 27, 2016^21^.

### Outcome variables

The outcome variable: was the fuel type used for cooking of the households in Ethiopia. These fuels were grouped into two categories in this study based on exposure to cooking smoke: "clean fuels" including electricity, liquid petroleum gas (LPG), natural gas, and biogas, and "solid fuels" including kerosene, coal/lignite, charcoal, wood, straw/shrubs/grass, and animal dung. Kerosene was considered in the solid fuels group in this study based on other previous studies on HAP that have reported kerosene as a polluting fuel^22–25^.then solid fuel was coded by 1, whereas clean fuel represented by 2.

### Explanatory variables

Covariates were classified into individual-level and community-level. Individual-level characteristics were the age of HHH (< 30, 30–40, 41–56, 56 +), sex of HHH (male, female), educational attainment (no education, incomplete primary, complete primary, incomplete secondary, Complete secondary, Higher), family size (< 3, 3–4, 5–6, 6 +), accessibility of electricity (yes, no), separate kitchen (yes, no), and household wealth index (lowest, second, middle, forth, highest) which were included in model 2. Community-level predictors were types of residence (rural, urban), region (capital city, large city, small city, town, and countryside), and community-level media exposure included in model 3.

### Data analysis

#### Spatial analysis

Spatial distribution, global spatial autocorrelation, spatial interpolation, and hotspot analysis of solid fuel use among households were done. First, the spatial distribution was observed using simple descriptive analysis, then the clustering effect using global spatial autocorrelation (Moran’s I) and hotspot regions of solid fuel use. This analysis was done using ArcGIS version 10.7 software.

To determine if the pattern of solid fuel was dispersed, clustered, or randomly distributed in the study area, the spatial autocorrelation (Global Moran's I) statistic was used. Details about spatial autocorrelation are published everywhere^26,27^. Local Moran’s I measure positively correlated (high-high and low-low) clusters and outliers. The statistical determination of cluster outliers is published everywhere^28^.

By computing Getis-OrdGi statistics for each area, it was possible to determine how spatial autocorrelation varied across the research location. To check that clustering was statistically significant, the Z-score was calculated, and the significance p-value was determined at 0.05 at the 95% confidence interval. If the Z-score is between − 1.96 and + 1.96, the p-value must be greater than 0.05 and vice versa. A cold spot is proclaimed if the p-value is less than − 1.96, whereas hotspot zones are announced if it is larger than + 1.96^29^.

#### Multilevel analysis

Since this study, the data source is hierarchal, multilevel logistic regression analysis with a random intercept at community, and individual levels was employed to determine the factors influencing the use of solid fuel for cooking. The multilevel modeling approach carries individual predictors at the individual and community levels and brings them into one analytical context. As well as they estimate variance partition between individual and community levels for understanding the relative importance of predicting variables to the outcomes variable at different levels^30^.

Model 0 (Null model) was fitted in the absence of independent variables to test random variability in the intercept and to estimate the mean odds ratio (MOR), intra-class correlation coefficient (ICC), and Proportion Change in Variance (PCV). Multilevel logistic regression (Model 1) examines the effect of individual factors and variables on solid fuel use. Whereas Model 2 examines the effect of community-level factors where Model 1 is nested in it. While model 3 is the combination of model 2 and model 3.

Variables with p-value < 0.20 in bivariable logistic regression were incorporated in multivariable binary logistic regression analysis. The results of this multilevel logistic regression analysis were explained using adjusted odds ratios (AORs) through 95% confidence intervals. The high-risk influences were recognized based on the p-value (p < 0.05) in the final model. All the statistical analyses were performed in STATA v 14.

Simple descriptive analysis, bivariate, and multivariate statistical analyses were performed in this study. Descriptive analysis was commenced to describe the frequency and percentage distribution. Akaike information criterion (AIC), Bayesian information criterion (BIC), and Bayesian deviance (− 2LLR) were used for model comparison. A model with a lower AIC, BIC, and deviance were preferred^31,32^.

#### Variance partition and model interpretation

The characteristics that make up the unobserved effect are probably correlated with variables included in the estimation. In such a large dataset, even if the sample of interest is randomly selected from a larger sample, the unobserved effect and included predictor variables are not independent^33^. Hence, the measures of community variation (random effects) were estimated as the intraclass correlation coefficient (ICC), variance partition coefficients (PCV)^34,35^, and median odds ratio (MOR) statistics were computed. The intraclass correlation coefficient (ICC) is a variance partition coefficient and can be calculated as the percentage of the community-level variance in the overall (both individual and community) variance^36^. A high ICC value indicates that the result difference comes more from the difference in community than in individuals^37^.

MOR can quantify unexplained cluster variability (heterogeneity) since ICC is with limitation. MOR can be understood as the median odds between two households using solid fuel for cooking, who are living in two communities with different levels of using solid fuel. A predictable understanding of the odds of individual-level forecasting variables can also be applied in MLR models to compare persons found within the same community^36^.

### Ethical approval and consent to participate

For this study, ethical approval was not required since this is a secondary analysis of the 2016 EDHS data. However, we registered and requested access to EDHS datasets from DHS online archive and received approval to access and download the data files. Consent to participate tot required since the study used secondary data.

## Results

### Characteristics of the study participants

Around seventy percent (69.5%) of the sampled household were from rural areas of the country. The mean age of a head of household is 43.05 ± (Std. Dev.16.494). Around one-fifth (23.02%) of households were exposed to community-level media while more than half (52.06%) of the participants were no education in educational status. A few (16.50%) of the households have separate rooms for kitchen and 36.14% of the households access electricity (Table 1).

**Table 1:** Descriptive statistics of the variables at individual and community levels, EDHS 2016.

Variable	Category	Sample population (%)	Frequency per variable (%)	
			Clean	Solid
Place of residence	Urban	5232 (31.42)	1462 (8.78)	3770 (22.64)
	Rural	11418 (68.58)	75 (0.45)	11,343 (68.13)
Age of HHH	< 30	4257 (25.57)	357 (2.14)	3900 (23.42)
	30–40	4132 (24.82)	388 (2.33)	3744 (22.49)
	41–56	4230 (25.41)	421 (2.53)	3809 (22.88)
	56 +	4031 (24.21)	371 (2.23)	3660 (21.98)
Mean 44.18 ± (Std. Dev. 16.221)				
Educational attainment (n = 16,592)	No education	8668 (52.06)	225 (1.35)	8443 (50.71)
	Incomplete primary	4071 (24.45)	275 (1.65)	3796 (22.80)
	Complete primary	587 (3.53)	91 (0.55)	496 (2.98)
	Incomplete Secondary	1299 (7.80)	215 (1.29)	1084 (6.51)
	Complete secondary	387 (2.32)	160 (0.96)	227 (1.36)
	Higher	1580 (9.49)	563 (3.38)	1017 (6.11)
Has the HH electricity	No	10,633 (63.86)	47 (0.28)	10,586 (63.58)
	Yes	6017 (36.14)	1490 (8.95)	4527 (27.19)
Sex of HHH	Male	11,413 (68.55)	962 (5.78)	10,451 (62.77)
	Female	5237 (31.45)	575 (3.45)	4662 (28.00)
Family size	< 3	6258 (37.59)	712 (4.28)	5546 (33.31)
	3–4	2647 (15.90)	290 (1.74)	2357 (14.16)
	5–6	4384 (26.33)	367 (2.20)	4017 (24.13)
	6 +	3361 (20.19)	168 (1.01)	3193 (19.18)
HH with separate kitchen room (n = 5788)	No	4833 (83.50)	442 (7.64)	4391 (75.86)
	Yes	955 (16.50)	262 (4.53)	693 (11.97)
Wealth index	Lowest	4676 (28.08)	4 (0.02)	6472 (28.06)
	Second	2348 (14.10)	6 (0.04)	2341 (14.07)
	Middle	2057 (12.35)	7 (0.04)	2050 (12.31)
	Forth	2020 (12.13)	19 (0.11)	2001 (12.02)
	Highest	5549 (33.33)	1501 (9.02)	4048 (24.31)
Region	Tigray	1734 (10.41)	120 (0.72)	1614 (9.69)
	Afar	1220 (7.33)	21 (0.13)	1199 (7.20)
	Amhara	1902 (11.42)	36 (0.22)	1866 (11.21)
	Oromia	1988 (11.94)	81 (0.49)	1907 (11.45)
	Somali	1564 (9.39)	12 (0.07)	1552 (9.32)
	Benishangul	1280 (7.69)	13 (0.08)	1267 (7.61)
	SNNPR	1897 (11.39)	25 (0.15)	1872 (11.24)
	Gambella	1280 (7.69)	17 (0.10)	1.263 (7.59)
	Harari	1135 (6.82)	208 (1.25)	927 (5.57)
	Addis Ababa	1489 (8.94)	854 (5.13)	635 (3.81)
	Dire Dawa	1161 (6.97)	150 (0.90)	1011 (6.07)

### The magnitude of using solid fuels

**Figure 1:**
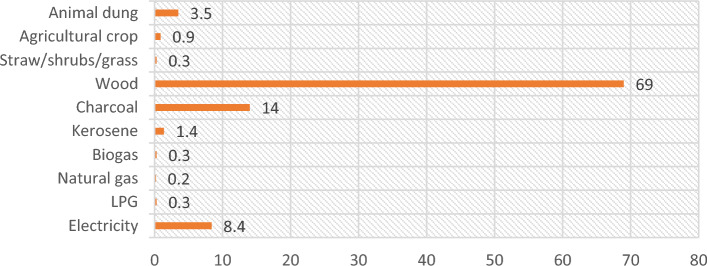
Components fuel type used by the households in percentage.

There are different types of fuel used for cooking reported by EDHS-2016 in the country. Amongst the solid fuel categories, wood was commonly (69%) reported by households followed by charcoal (14%) and very few (0.3%) only use LPG (Fig. 1).

**Figure 2:**
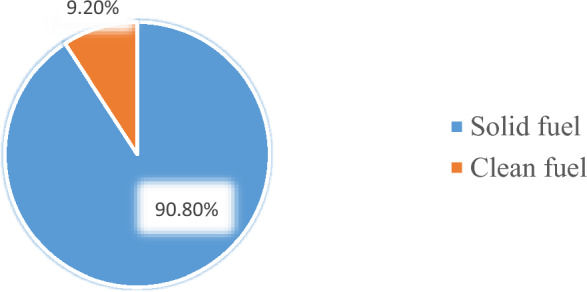
Solid and clean fuel use proportion of included households in Ethiopia, EDHS 2016.

The finding of this study revealed that most (90.8%) (95% CI (87.9%, 91.2%)) of the households depend on solid fuel use for cooking and heating (Fig. 2).

### Spatial distribution of solid fuel use in Ethiopia

**Figure 3:**
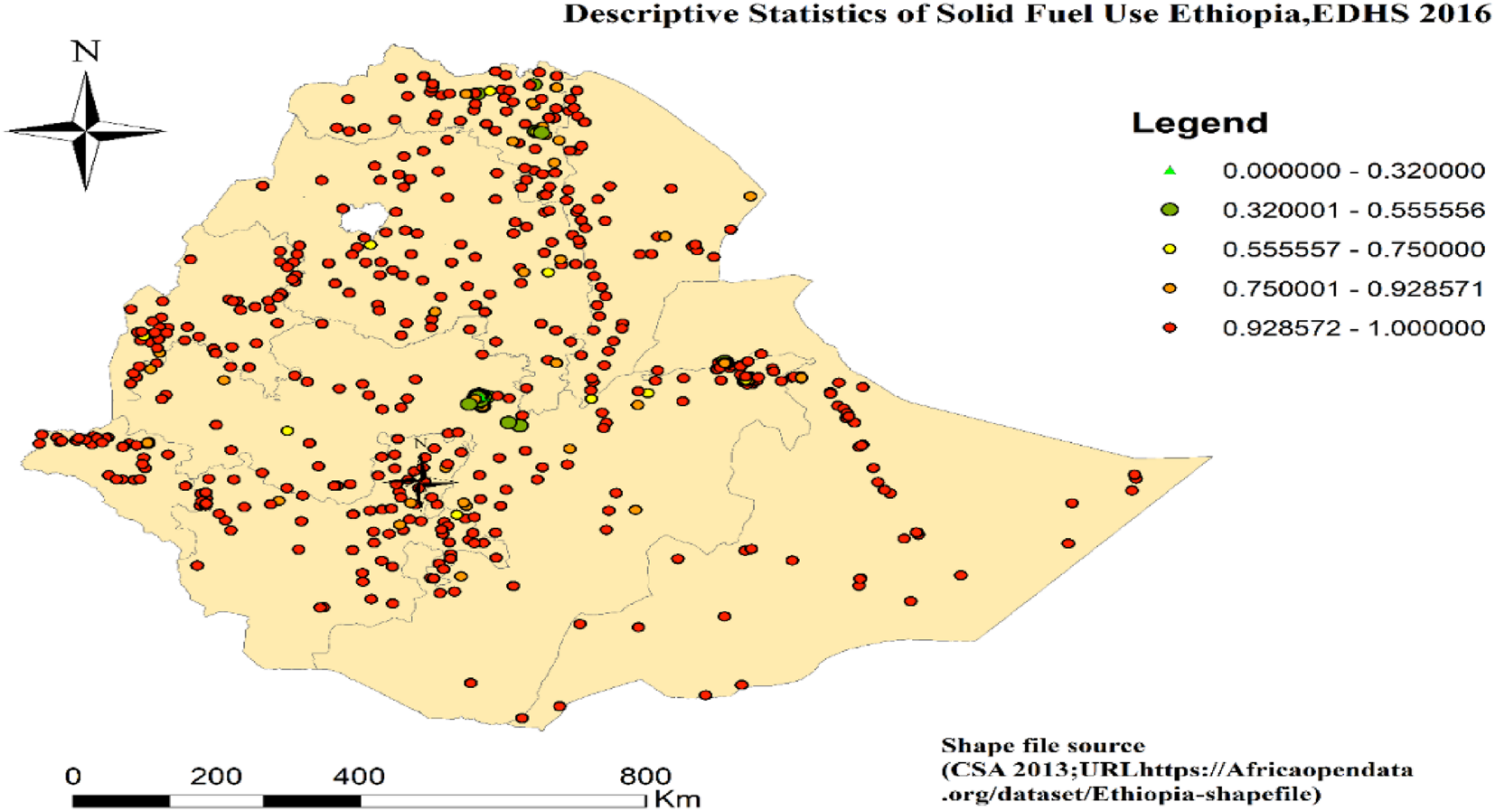
Spatial distribution of solid fuel use in Ethiopia (EDHS 2016).

The spatial analysis result of solid fuel use in Ethiopia revealed that the majority of the regions have concentrated cases of solid fuel use except for Addis Ababa and Dire Dawa (Fig. 3).

### Spatial pattern autocorrelation analysis of solid fuel use

**Figure 4:**
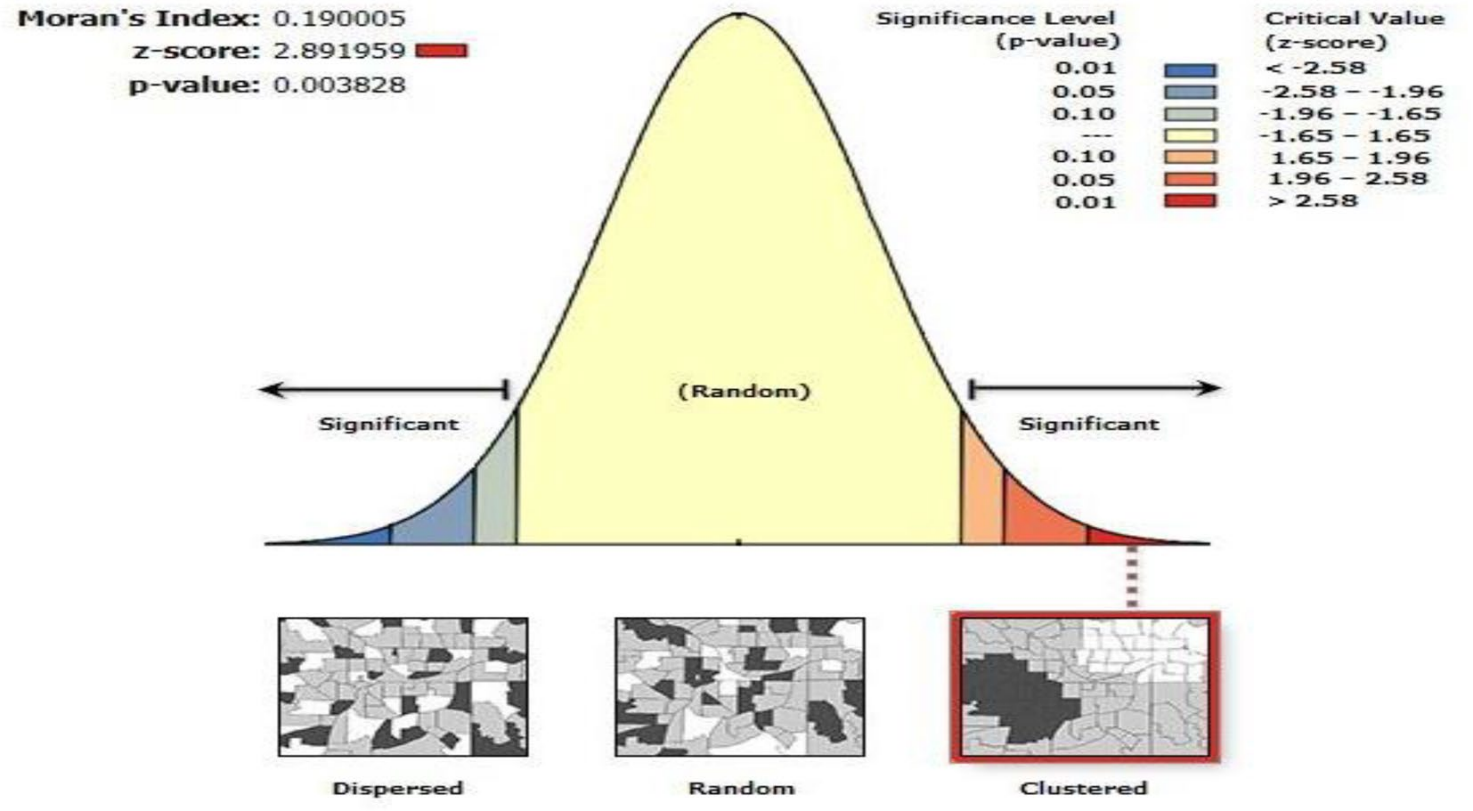
Spatial autocorrelation analysis of Solid Fuel Use in Ethiopia, 2016 EDHS.

The spatial distribution of solid fuel use among households in Ethiopia was spatially clustered with a Global Moran’s I value of 0.190005 (z-score = 2.891959, P-value < 0.003828) (Fig. 4).

### Cluster outlier analysis result of solid fuel

**Figure 5:**
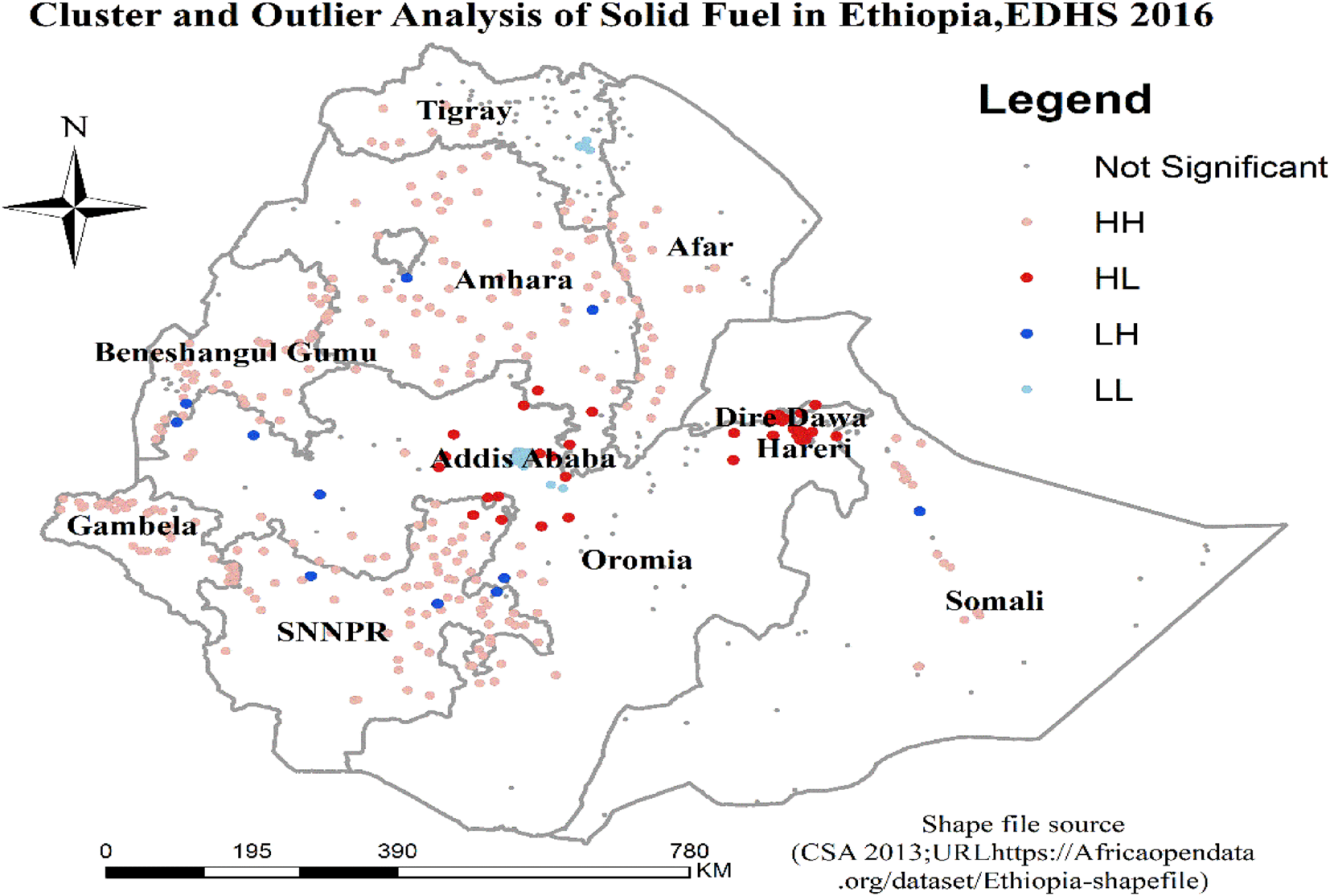
Cluster and Outlier Analysis of Solid Fuel Use in Ethiopia, 2016 EDHS.

The cluster outlier analysis result of solid fuel revealed that there are high outliers in Dire Dawa city administrative, Harari region, and Oromia region nearby Addis Ababa. On the other hand, low outliers were found in Western Oromia and Northeastern SNNPR (Fig. 5).

### Hot and cold spot areas for solid fuel use in Ethiopia

**Figure 6:**
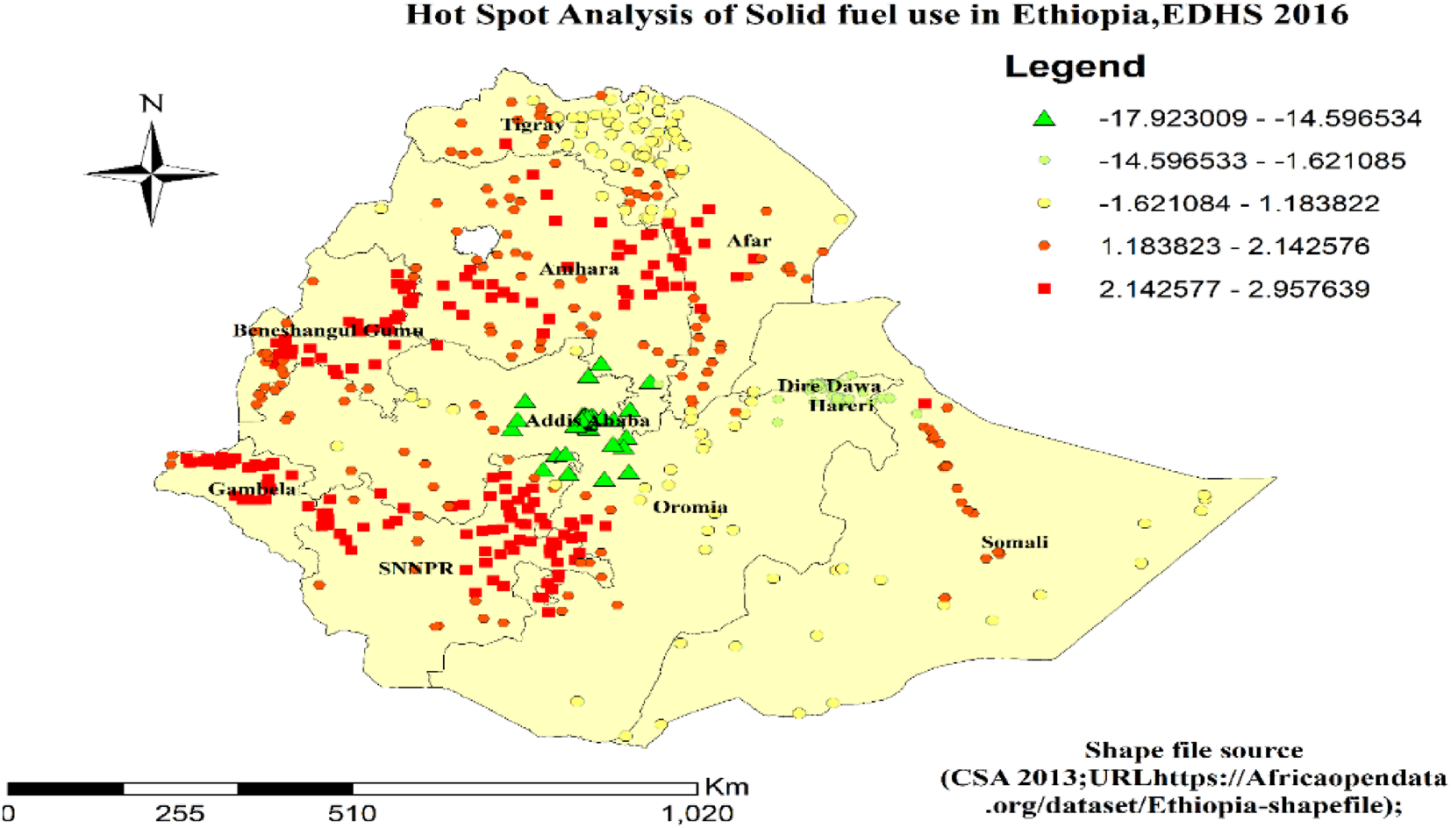
Hot and Cold Spot Analysis of Solid Fuel Use in Ethiopia, 2016 EDHS.

Local Getis-OrdGi analysis showed the extreme (hot and cold spots) locations of solid fuel use in Ethiopia. All parts of Gambella, Northern and Northeastern parts of SNNP, all parts of Benishangul, and all parts of Amhara region were identified as hotspot areas for solid fuel use. On the other hand, Dire Dawa and Addis Ababa city administrations were clod spot areas (Fig. 6).

### Factors associated with solid fuel use

Based on the final model, the sex of the household head, educational status of the mothers, place of residence, household wealth, household head's age, media exposure, and region were statistically significant factors associated with solid fuel use.

The current study indicated that ICC at the null model, model 1, model 2, and model 3 was 0.829, 0.393, 0.242, and 0.131, respectively.

Since it is statistically independent of the prevalence of the occurrence and can be easily calculated in the null model and more expanded models as follows MOR = $\mathrm{exp}[(0.95)\sqrt{Vc}$]. Therefore, the MOR value 29.96 in the null model revealed there was a variation in choosing solid fuel between clusters.

Proportional Change in Variance (PCV) indicated the proportion of the observed response variation that lies at each step of the model hierarchy. More than eighty-six percent (86.67%) of the variation in solid fuel use scores lies within the individual level, 95.24% is found within the community level, and 52.95% lies within the final hierarchy total variance. Thus, there is the highest variation in households' mean solid fuel use at the national level.

The lower values of the AIC, BIC, and Deviance values indicate fitted models. Therefore, the final model with the more complex variance components has the lowest AIC, BIC, and Deviance, 5479.181, 5656.745, and 5433.1812, respectively which indicated that model 3 was best fitted.

The sex of the household head was associated with using of polluting fuels (wood, straw/shrubs/grass, animal dung, crop residual, charcoal, coal, kerosene) for cooking. Male household heads were 1.38 (AOR 1.38, 95% CI (1.12–1.71)) times more likely to use clean fuel than female household heads.

This study also found that the odds of using solid fuels was 1.61 (AOR 1.61, 95% CI (1.20, 2.17)), and 1.49 (AOR 1.49, 95% CI (1.12, 1.99)) times more likely, for the age classes of < 30, and 30–40, respectively as compared with the class 54 + years as the reference group.

Women's education attainment also influences the household's primary cooking fuel choice. The odds of using solid fuel for cooking was 3.14 (AOR 3.14, 95% CI (1.13, 8.71)) among no education and primary education 2.16 (AOR 2.16, 95% CI (2.78, 5.96) times more likely as compared with the reference group of higher education.

The odds of using solid fuel 11.05 (AOR 11.05, 95% CI (5.68, 15.78)), 5.19 (AOR 5.19, 95% CI (5.43, 13.19)), 3.08 (AOR 3.08, 95% CI (2.44, 8.73)), and 1.30 (AOR 1.30, 95IC (1.07, 13.49)) was more likely for the wealth index classes of Poorest, Poorer, Middle, Richer respectively as compared with Richest as reference group.

Accessibility of electricity was the other statistically significant predictor of choosing the type of fuel for household consumption. Households not accessing electricity were 31.21 times more likely to use solid energy sources compared with those households having electricity (AOR 31.21, 95% CI (27.56, 42.67)).

The region was a significant community-level predictor variable in determining choosing type of fuel used. The odds of using solid fuel for cooking are 2.80 (AOR 2.80, 95CI (1.65, 4.77)), 2.58 (AOR 2.58, 95% CI (1.55, 4.32)) 4.02 (AOR 4.02, 95% CI (2.46, 6.55)) 14.40 (AOR 14.40, 95% CI (6.23, 21.15)) times more likely among households found in large city, small city, town, countryside as compared with the capital city.

The odds of using solid fuel for cooking (AOR 12.06, 95% CI (8.40, 17.32) was 12.06 times higher among rural households as compared with Urban households.

The other significant predictor of using solid fuel was community-level media exposure. The odds of using solid fuel increased with the households not experiencing community-level media exposure (AOR 6.00, 95% CI (4.61, 7.82)) (Table 2).

**Table 2:** Multilevel binary logistic regression analysis of individual and community-level factors associated with using solid fuel for cooking, EDHS 2019 (N = 16,650).

Variables	Model 0	Model 1 AOR (95% CI)	Model 2 AOR (95% CI)	Model 3 AOR (95% CI)
Individual level factors				
Sex of HHH				
Female		2.39 (1.98, 2.89)		1.38 (1.12, 1.71)*
Male^1^		1		1
Age of HHH				
< 30		2.60 (2.00, 3.37)		1.61 (1.20, 2.17)*
30–40		1.57 (1.22, 2.02)		1.49 (1.12, 1.99)*
41–54		1.43 (1.12, 1.53)		1.17 (0.86, 1.59)
> 54^1^		1		1
Family size				
< 3		1.18 (0.97, 1.45)		1.09 (0.87, 1.38)
3–5		1.80 (1.39, 2.34)		1.29 (0.96, 1.74)
≥ 6^1^		1		1
Educational status				
No education		8.85 (3.38, 23.21)		3.14 (1.13, 8.71)*
Primary		4.46 (1.71, 11.63)		2.16 (2.78, 5.96)*
Secondary		1.77 (0.66, 4.76)		1.47 (0.52, 4.20)
Higher^1^		1		1
HH with separate kitchen room				
Yes		6.21 (4.99, 7.73)		5.72 (3.54, 8.12)**
No^1^		1		1
Has the HH electricity				
Yes^1^		1		1
No		63.61 (19.91, 203.17)		31.21 (35.41, 42.67)**
Wealth index				
Lowest		14.73 (5.10, 17.56)		11.05 (5.68, 15.78)**
Second		11.95 (9.02, 31.18)		5.19 (5.43, 13.19)**
Middle		4.25 (2.52, 5.55)		3.08 (2.44, 8.73)*
Fourth		5.48 (4.44, 6.76)		1.30 (1.07, 13.49)*
Highest^1^		1		1
Community level factors				
Type of residence				
Urban^1^			1	1
Rural			8.10 (6.07, 10.80)	12.06 (8.40, 17.32)**
Media exposure				
No			17.10 (14.00, 20.90)	6.00 (4.61, 7.82)**
Yes^1^			1	1
Region				
Capital City^1^			1	1
Large city			7.36 (3.97, 13.63)	2.80 (1.65, 4.77)**
Small City			4.18 (2.29, 7.64)	2.58 (1.55, 4.32)**
Town			3.02 (1.90, 4.81)	4.02 (2.46, 6.55)**
Countryside			6.71 (3.94, 11.45)	14.40 (6.23, 21.15)**
Measures of variation of clustering				
ICC	0.829 (0.791, 0.862)	0.393 (0.334, 0.456)	0.242 (0.192, 0.299)	0.131 (0.094, 0.178)
Variance	16.003	2.1334	1.0480	0.4936
MOR	29.96	4.02	2.65	1.95
PCV	Ref	86.67%	95.24%	52.95%
Model fit statistics				
AIC	6612.281	5689.465	5958.778	**5479.181**
BIC	6627.721	5789.827	6059.14	**5656.745**
Deviance	6608.280	5663.4648	5932.778	**5433.1812**

*AIC* Akaike’s information criterion, *BIC* Bayesian information criterion, *ICC* intra-class correlation coefficient, *PCV* variance partition coefficients.

^1^Reference, **P-value < 0.001 (Adjusted OR), *P-value < 0.05 (Adjusted OR), HHH = household head, HH = household, Model 0 (Null model) was fitted without determinant variables; Model 1 is adjusted for individual-level variables. Model 2 is adjusted for community-level variables; Model 3 is the final model adjusted for both individual- and community-level variables. Significant values are in bold.

## Discussion

The primary objective of this study was to investigate spatial variation and predictors of household cooking fuel use for cooking in Ethiopia. Researchers have hypothesized that because solid fuel smoke contributes to local air pollution, neighbors can be harmed as well, even if they use clean fuels^38–41^.

The finding of this study showed that most (90.8%) (95% CI (87.9%, 91.2%)) of households used solid fuel as primary cooking and heating. In Sub-Saharan Africa, Southeast Asia, and the Western Pacific Region, the use of solid fuels prevails over cleaner fuel options, reaching 77%, 74%, and 74%, respectively^42^, which were lower than the result of this study. The finding of this study was higher than the finding from, Indian 76%^43^, Myanmar 79.0%^44^, and India 72%^45^ which of households use solid fuels as a primary source of energy for cooking.

This finding was similar to the result of the study done in Nepal 88%^46^ and South Africa 90%^47^, of which households used solid fuel for cooking and heating. The possible explanation for this variation could be because the sociodemographic, socio-economic-status, cultural, study period, and sample size are different among the study settings.

Solid fuel use appears to be more prevalent among households with female headship than in men-headed households. This finding was in line with the study done in Ouagadougou which showed that households headed by men have high socioeconomic status than woman-headed households^48,49^. Female household heads usually play a key role in household cooking decision-making activities^18^.

Solid biofuel choice as primary cooking fuel was statistically associated with the age of the household head. Which indicated that the odds of using solid fuels decreased as age increased. That is, respondents in the youngest age group use more solid fuel than participants in the oldest age study participants, in line with results from previous studies^50,51^. And this result contradicted another study that revealed increasing age and consumption of more dirty-burning fuels^5^.

The wealth index was a predictor variable that significantly determined the type of cooking fuel used in a household. Previous Similar studies from Cameroon, and India described that household wealth governs the change in energy sources from solid fuel use to clean for domestic purposes^52,53^. This might be due to as household income increases, households tend to use processed energy and more efficient fuels for cooking, lighting, and heating^54,55^ and poorer households are likely to stick with solid fuels for cooking since clean energy cost is high. The richest people are expected to have market access and the capability to own an improved stove^56^.

Another sociodemographic significant factor is the education status of mothers. The odds of using solid fuel type were higher among women with lower educational attainment. This finding was supported by other studies which indicated that increases in educational level increase the chance of consuming cleaner energy as the central source of cooking fuel^57,58^. These all show that through education families possibly become informed on the advantages of using cleaner fuels and the problems regarding solid fuel type^1^. Or access to education is important for endorsing consciousness of clean energy, non-polluting fuels, better cook stoves, and the health implications of using solid fuels for cooking^1^. Similar findings showed that households with more educated mothers are more likely to choose cleaner fuels since they understand the impact of using solid fuels. Due to a matter of fact the more the woman has attained high education, the more the likelihood that her husband has accomplished high schooling level too^48,59^. Therefore, there is the synergetic effect of education that would lead to a certain level of understanding and perception of the risk of using solid fuel^60,61^.

According to the finding of this study, there was a statistically significant association between accessing electricity and the type of fuel used for cooking in households. This finding was supported by other studies done previously^62–67^. This might be due to the price of electricity, which is relatively high compared to other fuel types, or due to the inconsistent nature of the power supply in the country. Therefore, the undesirable effect for electricity prices put forward that the subsequent consequence of a price escalation is toward low-grade fuels selection.

The odds of using solid fuel among the households having separate rooms used as the kitchen was higher than the households not having separate rooms used as kitchen counterparts. This might be due to using separate kitchen locations for cooking would reduce the problem related to using solid fuel. Therefore, even other factors are not substantial effect on choosing solid fuel for cooking; households may prefer low-cost solid fuel types since they have separate kitchen locations. This finding was in line with the study done in Ouagadougou^48^.

Family size was not a statistically significant predictor in choosing a fuel type for cooking. However, other previous similar studies found that wood as an energy source is by far the fuel of choice for a majority of households with comparatively representative sample sizes^68–70^.

The finding of this study revealed that there was a statistically significant association between the place of residence and the kind of fuel used. Living in urban residences increases the chance of using clean fuel types like electricity and LPG. This result was consistent with other similar studies in different parts of the world, those examining the association between place of residence, and choice of cooking fuel type^63,71,72^. The possible explanation, for this finding, is solid fuels for cooking in rural regions because alternative clean fuels cannot be accessible for households consumptions, and some biomass fuels are everywhere in the nation, such as straw, animal dung, crop residue, and wood^73^. The other opinion based on this finding, leaders within the urban community might also influence the adoption of using clean fuel since solid fuels are aesthetic problems.

Another main result of this study was that the odds of using solid fuel increased with the households not experienced community-level media exposure. This might be due to that participants not exposed to different media may not know about health problems related to using solid fuel for cooking and heating. Therefore, they used more solid fuels at large than media-exposed respondents.

The Global Moran’s I value indicated that spatial distribution of solid fuel use among households in Ethiopia was spatially clustered than random. Following this value, Local Getis-OrdGi analysis was done to show the extreme (hot and cold spots) locations of solid fuel use in Ethiopia. Therefore, all parts of Gambella, Northern and Northeastern parts of SNNP, all parts of Benishangul, and all parts of Amhara region were identified as hotspot areas for solid fuel use. The distribution of household types of cooking fuel differs through the regions found in Ethiopia, reflecting the variances in natural resource endowment across regions, also the level of economic development, the degree of urbanization, and fuel type accessibility. The use of solid fuel for cooking is primarily strongly associated with households in the countryside, which implies that solid fuel can be easily accessed in such areas. This study is with some strengths and limitations. The key strength of this study was that the data was gained from urban and rural areas, which means the data is a nationally representative survey. The survey covered both. The first limitation, the EDHS did not show the clear classification of cooking fuels into solid and clean fuels. Secondly, there is the possibility of recall bias since the data collection is through self-reported interviews and social desirability bias.

## Conclusion

This finding revealed that a large proportion of households in Ethiopia heavily depend on biomass, especially wood, for cooking. Estimating that a large proportion of the population is dependent predominantly on solid fuel use for cooking. The statistical analysis showed that access to electricity, educational attainment of the mothers, wealth index, sex and age of household, and place of residence had a significant role in determining the type of cooking fuel among households. There was significant spatial heterogeneity in solid fuel use for cooking in Ethiopia. Implementing major policy interventions should be introduced to reduce solid fuel use for cooking and inequalities of accessing clean fuel in Ethiopia.

## Data Availability

The data are available online from www.measuredhs.com.

## Abbreviations

AIC: Akaike information criterion
AOR: Adjusted Odds Ratio
CI: Confidence interval
DHS: Demographic and health survey
EAs: Enumeration areas
EDHS: Ethiopian demographic and health survey
ICC: Intra class correlation
MOR: Median odd ratio
PCV: Proportional change in variance
